# Early hearing threshold changes and peculiarities of audiometric assessments among patients in a drug-resistant tuberculosis treatment center

**DOI:** 10.4314/ahs.v21i1.30

**Published:** 2021-03

**Authors:** Olusola A Sogebi, Bolanle O Adefuye, Ebenezer A Ajayi

**Affiliations:** 1 Department of Surgery, Faculty of Clinical Sciences, OACHS, Olabisi Onabanjo University, Sagamu, Nigeria; 2 Department of Medicine, Faculty of Clinical Sciences, OACHS, Olabisi Onabanjo University, Sagamu, Nigeria; 3 Sacred Heart Hospital, Lantoro, Abeokuta, Nigeria

**Keywords:** Early hearing threshold changes, audiometric assessments, tuberculosis treatment center

## Abstract

**Background:**

Hearing threshold changes occurred relative to baseline at both one and two weeks after onset of aminoglycoside therapy.

**Objectives:**

To assess changes in audiometric hearing thresholds between pre-treatment values and two weeks into therapy. To document observed changes, and occurrence of ototoxicity within the period.

**Methods:**

Prospective analytical cohort study on drug-resistant tuberculosis patients. Basic demographic parameters were taken. Three-point audiometric assessments within two weeks into therapy were done. Percentage of patients with ototoxicity were calculated. Pure tone threshold changes between the three audiometric values were compared.

**Results:**

Audiograms of 53 patients comprising 56.6% males; age range was 13 to 91 years. Both air and bone conduction hearing thresholds significantly worsened between baseline and one week into therapy (p=0.011, and 0.015 respectively), and between baseline and two weeks into therapy (p=0.003 and 0.042 respectively). Minimal insignificant reduction occurred between both air and bone conduction hearing values of week 1 and week 2 of therapy (p= 1.000 and 0.856 respectively). By audiometric criteria, 4 patients (7.5%) developed ototoxicity within two weeks of treatment.

**Conclusion:**

Audiometric assessments within two weeks into therapy with anti-tuberculous therapy may not represent baseline audiometry. 7.5% of the patients developed ototoxicity within two weeks of therapy.

## Introduction

Pure tone audiometry is the simplest subjective audiological assessment of hearing levels in adults. Audiometric assessments are employed in serial monitoring of hearing levels in patients that are predisposed to worsening of hearing by virtue of occupation or administration of potentially ototoxic medications. The former include subjects that work in industries where there is exposure to unduly loud noise produced by high powered machinery, road traffic officers, workers at airports, motor-parks and markets.^1^,^2^

The other at-risk group include patients on prolonged intake of potentially noxious medications like aminoglycosides such as patients with cystic fibrosis, pseudomonas aeruginosa infections, neonatal necrotizing enterocolitis, and those with drug-resistant tuberculosis. 3 Ototoxicity is a major side effect of prolonged use of aminoglycosides, and it has led to relative restriction of its use in developed countries, limiting its indications to very specific or severe infections. The use of aminoglycosides has however remained less restrained in the less developed countries because of its broad spectrum antimicrobial activity including activity against aerobic Gram negative organisms, its bactericidal action, relatively low cost, and its hypoallergenic properties.^4^ The World Health Organization (WHO) actually specified use of aminoglycosides as second line drugs for treatment of tuberculosis.^5^

Ototoxicity may be dose-dependent effect which tends to occur with accumulation of drugs over a duration of time leading to permanent hearing threshold shift and hearing loss.^6^ Thus serial monitoring of hearing assessment in patients on aminoglycosides therapy becomes expedient. The baseline audiometry is the first audiometric hearing assessment, usually performed before exposure to a potentially noxious agent or before commencement of therapy, to which subsequent audiometric assessments are compared to detect ototoxicity based on specific criteria.^7^

In tuberculosis treatment-center, it may sometimes not be feasible to perform the first audiometric assessment on patients before commencement of medications, and the assessments are performed as soon as possible within two weeks of commencement of therapy. This first assessment is thus still regarded as baseline audiometry, on the assumption that significant hearing threshold changes would not have occurred within this period of time. Researchers however query this assumption and insist that only pre-therapy audiometric assessment can represent baseline audiometry. Some authors have insisted that high frequency hearing loss (ototoxicity) can occur within two weeks of commencement of patients on aminoglycosides.^8^,^9^ Clarification of the controversy on what represents baseline audiogram becomes expedient since it indirectly determines occurrence of ototoxicity, which is an irreversible hearing impairment.

This study thus assessed if the hearing threshold of patients changed significantly between the pre-treatment values and those within the first two weeks of therapy. The changes ascertained if audiogram taken within the first two weeks of therapy could represent baseline audiometry or not. The study also noted the pattern of such changes, and clarified possible occurrence of ototoxicity within the two-week period. Lastly the challenges and peculiarities of serial audiometric assessments in our center which is less than an ideal setting, were addressed.

## Patients and methods

The study is a prospective analytical cohort study employing consecutive patients with diagnosis of drug-resistant Tuberculosis who were admitted for intensive phase of treatment at Ogun state Drug-resistant Tuberculosis treatment center, domiciled at the Sacred Heart Hospital (Special), Abeokuta, Nigeria. The patients were admitted from February to August 2018. Ethical approval to conduct the study was granted by the Ethics Committee of Sacred Heart Hospital, Lantoro, Abeokuta. Patients were diagnosed as DRTb based on demonstration of mycobacterium resistance to Rifampicin and Isoniazid by bacteriological tests Gene-Xpert MTB/Rif assay and drug susceptibility test, DST. At initial evaluation of the patients, age and sex of patients were recorded, and their ears were examined among other systems. There was no obvious nor suspected ear pathology that could rapidly progress to hearing loss in the patients. Thereafter hearing level evaluations were performed before commencement of therapy (Baseline), then at one, and two weeks into anti-tuberculous therapy regimen, which contained an aminoglycoside (Kanamycin) as a major component. The drug dosage was based on WHO recommendations which was adopted for implementation by the National Tuberculosis program of Nigeria in 2017. Specifically for Kanamycin, dosage is 15mg/kg body weight (maximum of 1gm); for adults over 59 years of age, the dose will be reduced to 10mg/kg body weight (maximum dose 750mg). 10 The drug should normally be given as a once daily dose for the entire four months of intensive therapy.

All eligible and consenting patients were included in the study. Patients excluded from the study were those who had previously been on intensive phase of treatment in the community prior to admission at the center, patients with audiometric evidence of profound hearing loss at the baseline, and those who could not complete i.e. have the three audiometric assessments within the stipulated two weeks.

Pure tone audiometry was performed using a calibrated hybrid diagnostic audiometer, Interacoustics AD 226 (Interacoustics A/S audiometer, Aile 1.5500 Middlefart, Denmark, 2016) in a quiet room with ambient noise level of 27dB SPL, using DD45 audiometry headset for the air conduction, and B71bone conductor for the bone conduction. PTA was performed by a Consultant Otorhinolaryngologist, according to the standards of audiometric assessments. Test frequencies were 0.25, 0.50, 1.0, 2.0, 4.0, and 8.0 kHz for air conduction, and 0.25, 0.50, 1.0, 2.0, and 4.0 kHz for bone conduction. From the audiographs, mean (average) threshold shifts for each ear, and the hearing average (pure tone average) for both ears were calculated for the air-conduction and bone-conduction hearing thresholds in each patient at the three different time points. Ototoxicity was assessed by comparing air-conduction hearing thresholds of latter audiograms with the baseline audiogram for hearing threshold changes for each patient. Any change observed on the audiogram was subjected to a retest the same day. The American Speech-language Hearing Association (ASHA) criteria for detection of ototoxicity^7^ was adopted defined by >20 dB pure-tone threshold shift at one test frequency, or >10 dB shift at two consecutive test frequencies, or threshold response shifting to “no response” at three consecutive test frequencies. The percentage of patients that fulfilled the criteria for ototoxicity was calculated.

The changes that occurred in the pure tone averages within the periods were assessed with repeated measures of Analysis of Variance (ANOVA) using the SPSS version 20. Results were presented in tabular and graphical formats, with statistical significance set at p<0.05.

## Results

There were seventy patients that were admitted during the study period, with 53 patients completing the audiometric assessments from baseline (pre-treatment) to two weeks into treatment. Data of seventeen patients were excluded comprising 7 patients that had commenced intensive phase of therapy from the community, 8 patients who became acutely sick and weak within the two weeks on medications, thus could not complete the three audiometry assessments and 2 patients who died within two weeks on therapy.

There were 30 (56.6%) males, and age ranged from 13 to 91 years. The age distribution in relation to the sex of the patients is shown in Table 1.

**Table 1:** Age distribution according to Sex of the Patients

Age group (Years)	Male (%)	Female (%)	Total (%)
11–20	1 (1.9)	2 (3.8)	3 (5.7)
21–30	8 (15.1)	7 (13.2)	15 (28.3)
31–40	4 (7.5)	4 (7.5)	8 (15.1)
41–50	9 (17.0)	6 (11.3)	15 (28.3)
51–60	7 (13.2)	4 (7.5)	11 (20.8)
61 and above	1 (1.9)	0 (0.0)	1 (1.9)
Age range	20–91	13–58	13–91
Mean ±SD	41.7 ±16.2	37.0 ±12.9	39.7 ±14.9

Table 2 shows the mean hearing threshold shifts in each of the ears is about 5 dBHL, within the first and the second week of treatment. The mean air-conduction hearing thresholds over the period is also depicted. Mauchly's test confirmed sphericity of the data; Mauchly's W 0.998, ≈ x^2^ 0.124, >df 2, sig 0.940. A repeated measure ANOVA discovered that mean air conduction thresholds differed significantly between time points F (2,104) =7.010; p=0.001. Post-hoc test of pairwise comparisons using the Bonferroni adjustment revealed air conduction hearing thresholds increased between the baseline and one week into therapy (32.537 ±10.679 Vs 34.892 ±10.992), between the baseline and two weeks into therapy (32.537 ±10.679 Vs 34.694 ±10.896) which were both statistically significant (p=0.011, and p=0.003 respectively). There was minimal worsening between hearing threshold values of week 1 and week 2 of therapy which was not statistically significant (p=1.000).

**Table 2:** Air conduction hearing threshold changes in the patients

**Mean threshold shifts**	**Right ear**	**Left ear**
Week 1	5.128 ± 3.152	5.318 ±3.314
Week 2	5.642 ±3.233	5.711 ±3.731

**Period**	**Mean hearing threshold**	**95% confidence interval**
Baseline	32.537 ±10.679	29.304–35.191
Treatment Week 1	34.892 ±10.992	31.863–37.922
Treatment Week 2	34.694 ±10.896	31.691–37.698

**Figure 1:**
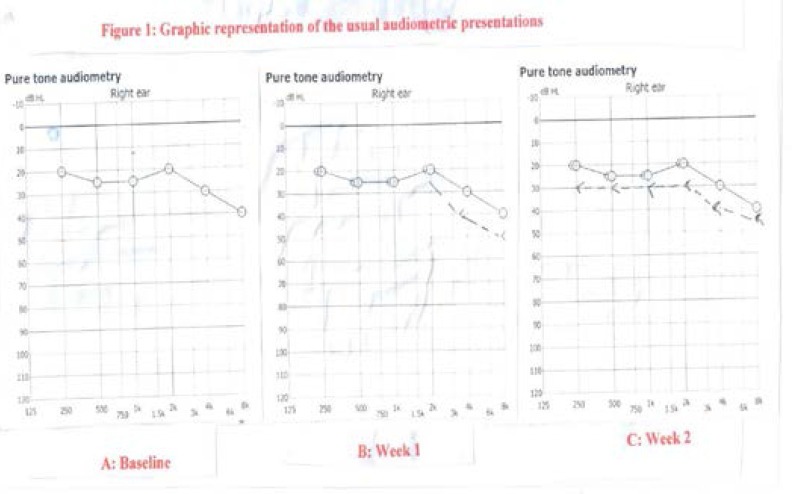
Graphic representation of the usual audiometric presentations

A graphic representation of the usual presentation of audiometry for baseline, week 1 and week 2 is depicted in Figure 1.

The same trend of hearing threshold shifts was observed in the bone conduction patterns of the patients shown in Table 3 which displays the average hearing thresholds shifts in each of the ears of about 4 dBHL, within the first and the second week of treatment. The mean hearing threshold values within the two weeks are also shown. Sphericity of the data was confirmed; Mauchly's W 0.903, ≈ x^2^ 5.221, df 2, sig 0.074. Repeated measure ANOVA revealed that mean bone conduction thresholds differed statistically significantly between time points F (2,104) = 5.432; p=0.006. Bonferroni adjustment in Post-hoc test of pairwise comparisons showed bone conduction hearing thresholds increased between the baseline and one week into therapy (25.670 ± 8.312 Vs 28.500 ± 9.464), between the baseline and two weeks into therapy (25.670 ± 8.312 Vs 29.477 ± 8.344) which were both statistically significant (p=0.015, and p=0.042 respectively). There was minimal worsening between hearing threshold values of week 1 and week 2 of therapy which was not statistically significant (p= 0.856).

**Table 3:** Bone conduction hearing threshold changes in the patients

**Mean threshold shifts**	**Right ear**	**Left ear**
Week 1	4.230 ± 3.993	4.182 ±3.814
Week 2	4.803 ±3.382	4.511 ±3.121

**Period**	**Mean hearing threshold**	**95% confidence interval**
Baseline	25.670 ± 8.312	22.039–28.621
Treatment Week 1	28.500 ± 9.464	25.891–31.109
Treatment Week 2	29.477 ± 8.344	27.178–31.777

By audiometric criteria, no patient developed ototoxicity within the first week of treatment, while 4 patients (7.5%) developed ototoxicity within two weeks of treatment.

## Discussion

The findings of this study revealed that there were significant changes between the hearing thresholds pre-treatment (baseline) and those taken after commencement of therapy, for both the air and bone-conduction thresholds in drug-resistant tuberculosis patients on aminoglycosides. Thus audiograms taken after commencement of therapy do not represent baseline audiometry. The criteria for confirmation of DRTb had been stated, although it was difficult to clarify if the drugs previously taken by the patients were ototoxic. However with the insistence of WHO on the use of aminoglycosides only as a second line drug in the treatment of tuberculosis, it is doubtful if any patient would have received aminoglycoside as a first line of treatment medication. Thus the audiometric hearing changes observed could reasonably be attributed to the aminoglycosides therapy received by the patients within the two weeks.

Ototoxicity is characterized by increased hearing thresholds, or observed worsening in hearing over a period of time. In our patients, we envisaged hearing levels could deteriorate after commencement of aminoglycosides therapy.^11^ This study found that patients had hearing threshold shifts and hearing levels worsened significantly within the first and second weeks of therapy compared to the pre-treatment values. However, there were no appreciable changes in the hearing levels between the first and second week on therapy. Few studies had observed that hearing thresholds of patients were not adversely affected by injection of aminoglycosides notably Gentamicin alone, or in combination with steroids, especially Dexamethasone in patients with intractable Meneire's disease.^12^,^13^ Dose-dependent effect of aminoglycosides has been reported previously,^14^ however a general trend of worsened hearing acuity within the first week on medications has not been established.

In tandem with the general trend of worsened hearing thresholds noticed within the first two weeks of therapy, four (7.5%) of our patients met the audiometric criteria for ototoxicity within the first two weeks on therapy with aminoglycosides. We regarded these patients as having early-onset ototoxicity. Ideally to determine if changes meeting the ASHA criteria for ototoxicity were related to true hearing changes or simple bidirectional variability in responses, post baseline air conduction threshold changes meeting the ASHA criteria in the reverse direction should be performed.^15^

While the general tendency of prolonged injection of aminoglycosides in producing ototoxicity has been acknowledged, individual risk factors, disease, and genetics that influence susceptibility in a patient population are likely to obscure a dose-response relationship.^16^ Danhauer et al,^17^ had noted that there was a trend for patients with abnormal initial (baseline) audiograms to develop further evidence of hearing impairment. Some people have an inherited predisposition that renders them highly sensitive to the ototoxic effects of the antibiotics. 18 In such people, aminoglycosides taken at levels that are well within the therapeutic range can result in rapid, profound, and irreversible hearing loss.^19^ Even a single dose in a predisposed individual can result in permanent hearing loss.^19^,^20^

Aminoglycoside-induced and non-syndromic deafness has been shown to have a genetic susceptibility and the pathogenic mitochondrial 12S rRNA A1555G mutation was identified as the primary factor underlying the hearing loss in many familial as well as in genetically unrelated cases.^21^ This phenomenon has been documented to occur in a variety of ethnic groups, on a variety of mitochondrial backgrounds, and in populations around the globe.^22^ It may be assumed that the patients that had early onset ototoxicity probably had some genetic predisposition. It might not be practicable to perform genetic screening on all patients who were to be on prolonged aminoglycosides therapy in our environment, however a suggestion that these patients already identified for early-onset ototoxicity be screened with molecular tests for gene mutations is reasonable. Casano et al,^23^ emphasized the clinical relevance of taking a family history of ototoxicity before administering aminoglycosides to any patient. Such history may be difficult to elicit in a population where aminoglycosides and other medications are relatively unregulated, when patients are unaware of medications they receive, and where pharmaco-vigilance is low.

Some peculiarities of audiometric assessments in our local setting that caused delay in audiometric assessments of our patients must be addressed. Three factors could be attributable to this delay namely; low level of awareness of the caregivers on the importance of audiological assessment of patients before commencement of therapy, non-availability of calibrated diagnostic audiometer and its accessories, and dearth of appropriately-trained technical manpower.

Level of awareness and knowledge of medical personnel concerning management of DRTb including detection of ototoxicity in patients on aminoglycosides usage should be high. We have initiated training and re-training the caregivers of patients on the protocol for appropriate treatment for DRTb by regular attendance of workshops and seminars. Recently the challenge of procuring the necessary audiological equipment for the DRTb treatment center was solved with the supply of a hybrid diagnostic audiometer which can be powered either via the public electricity grid or by dry cell battery. This equipment has also solved the problem of unstable and erratic power supply and made it possible to investigate patients as soon as possible on their arrival at the treatment center, before commencement of therapy. More recently, a stand-by medical personnel who can perform Pure Tone Audiometry on patients without delay has been employed. The lingering challenge is absence of a sound-proof audiometric booth in the center at present. However the audiometry is performed in a quiet room within the center.

Some other challenges in managing the patients exist. Experts recommend that for monitoring of ototoxicity in patients on medications, audiometric assessments must be performed prior to commencement of treatment and once or twice a week for the period of therapy.^24^ However the programmatic management of patients as operated at the center recommend baseline audiometry is performed before commencement of therapy, and at monthly intervals while the patient is on admission for the intensive phase of therapy.^25^ This practice allowed a lacuna which prevented ototoxicity from being detected earlier than one month into therapy. Findings of this study has demonstrated that ototoxicity can occur earlier than the first month of therapy in our patients.

Ototoxicity tends to affect the high frequencies first and then the lower frequencies. 6,26 It has thus been suggested that for detection of ototoxicity, attention should be focused more on the higher frequencies above 8kHz, thus ultra-high frequencies ranging from above 8kHz up to 20kHz needed to be measured.^27^ The conventional audiometer assesses frequencies up to a maximum of 8kHz, and the ultra-high frequencies are not measured, limiting diagnosis of ototoxicity in our patients. The provision of an audiometer which can measure the ultra-high frequencies in our patients is anticipated.

Another peculiarity in our center is related to the patients. Many patients, especially those that have developed ototoxicity or having vestibular or otologic symptoms, develop apathy towards repeated audiometric assessments. This is not surprising because they feel frustrated with some of the symptoms including hearing impairment, tinnitus, and sometimes distortions in balance, which are often irreversible, nor amenable to further medication.^28^ Clinical practices and studies use the Dizziness Handicap Inventory and Tinnitus Handicap Inventory to monitor for those side effects, as they are easy to administer and inexpensive.^29^ The main treatment we offered for hearing impairment is amplification of sounds using the hearing aid. Unfortunately, hearing aids are not fitted early which compounded patient's frustration.

It is noteworthy that despite our setting being “less than ideal”, the clinical services provided in our center is rather typical of what obtains in many parts of our country and other countries in sub-Saharan Africa. While this study has been able to clarify some issues concerning audiometric assessments in our environment, its main limitation is that the findings are not generalizable for all patients on prolonged aminoglycosides therapy. Although it was confirmed that the patients had tuberculosis which was resistant to rifampicin, other medications that the patients previously received could have been remotely ototoxic. The initial assessment could have been baseline audiometry before aminoglycoside treatment, but none of the baseline audiometry had evidence of ototoxicty. The fact that genetic screening could not be done on patients that developed ototoxicity is also a limitation.

## Conclusion

The study found that audiometric assessments done within two weeks of commencement of therapy for patients on prolonged aminoglycosides anti-tuberculous therapy do not represent baseline audiometry. Less than one tenth (7.5%) of the patients developed early -onset ototoxicity within two weeks of therapy.
